# Optical control of spin-polarized photocurrent in topological insulator thin films

**DOI:** 10.1038/s41598-018-33716-0

**Published:** 2018-10-18

**Authors:** Hiroaki Takeno, Shingo Saito, Kohji Mizoguchi

**Affiliations:** 1 Department of Physical Science, Osaka Prefecture University, 1-1 Gakuen-cho, Naka-ku, Sakai, Osaka 599-8531 Japan; 2 National Institute of Information and Communications Technology, 4-2-1 Nukui-Kitamachi, Koganei, Tokyo 184-8795 Japan

## Abstract

Dirac electrons in topological insulators (TIs) provide one possible avenue to achieve control of photocurrents and spin currents without the need to apply external fields by utilizing characteristic spin-momentum locking. However, for TI crystals with electrodes it is actually difficult to characterize the net flow of spin-polarized photocurrents because of the coexistence of surface carriers and bulk carriers generated by optical excitations. We demonstrate here that the net flow directions of spin-polarized photocurrents in TI polycrystalline thin films without electrodes can be precisely and intentionally controlled by the polarization of the excitation pulse alone, which is characterized by performing time-domain terahertz (THz) wave measurements and time-resolved magneto-optical Kerr rotation measurements that are non-contact methods. We show that the amplitudes of s-polarized THz waves radiated from photocurrents under right- and left-circularly polarized excitations are inverted relative to one another. Moreover, we observe the inversion of time-resolved magneto-optical Kerr rotation signals between the two excitations. Our results will open the way as innovative methods to control spin-polarized electrons in optoelectronic and spintronic TI devices without the need to apply external fields.

## Introduction

Topological insulators (TIs) are attractive materials in looking toward the next generation of electronic devices because there exist Dirac electrons on their surface. The spin polarization of these Dirac electrons is locked in the direction perpendicular to the momentum, which is referred to as spin-momentum locking^1–3^. When TIs are irradiated with circular polarized light, electrons in TIs are excited from a bulk state or surface state to higher surface states according to optical selection rules, including spin selection^4,5^. Therefore, it is expected that photocurrents will flow without any need to apply external electric fields, due to asymmetric excitations in the momentum space^5^. In addition, it is anticipated that spin currents are simultaneously induced with the generation of photocurrents in TIs, owing to the spin-momentum locking. Hence, the flow directions of spin-polarized photocurrents in TIs might be readily controlled by varying the polarization of the excitation light.

For TI crystals with electrodes, light irradiation on the sample induces a bulk thermoelectric current due to the Seebeck effect^6,7^ and a photocurrent that is dependent on the crystal axes in the surface layer^8–10^. While the flow direction of the former current depends on the thermal distribution between the electrodes, the flow direction of the latter can be modified by rotating the sample in-plane. Both currents influence the primary flow directions. Therefore, it is difficult to estimate not only the net flow direction of the photocurrent alone but also the relationship between the flow direction of the photocurrent and spin polarization in TIs with electrodes. In order to realize the precise photocurrent control with the polarization of excitation light, we have measured the terahertz (THz) waves radiated from TI polycrystalline thin films without electrodes, which gives us the information about the dynamics of photo-excited carriers, because the electric fields of the THz waves radiated from photocurrents due to photo-excited carriers are proportional to the time derivative of the photocurrents^11^. In the present study, we demonstrate that the amplitudes of the THz waves radiated from the photocurrent flowing in the sample depend on the polarization of excitation pulses, which indicates the potential to precisely and intentionally control the flow directions of the photocurrents generated at the topological surface state by the polarization of excitation pulses. On the other hand, the photocurrent is expected to be spin-polarized due to spin-momentum locking. The spin polarization of photo-excited electrons with and without an applied external magnetic field has been investigated in previous studies^12–14^. In this study, we investigated the spin polarization of the photocurrent by measuring time-resolved magneto-optical Kerr rotation signals^15–17^ without any external magnetic field. We show that optical control in terms of the spin polarization of photo-excited carriers in TI thin films can be achieved.

## Results

### THz wave measurements

**Figure 1:**
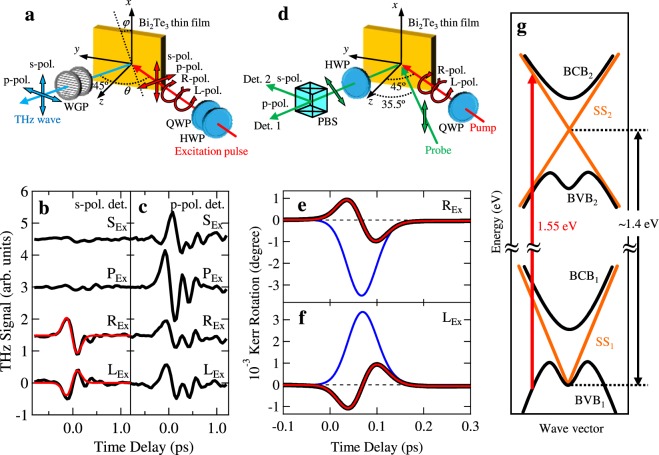
THz waveforms and Kerr rotation signals due to spin-polarized photocurrents generated in Bi_2_Te_3_ TI thin films by excitation pulses with various polarizations. (**a**) Schematic of optical setup of THz wave measurements. Excitation pulses from a Ti: sapphire pulse laser with the center wavelength of 800 nm are focused on the Bi_2_Te_3_ thin film at an incident angle of *θ* (=45°). *φ* indicates the azimuthal angle around the *z* axis. The polarization of the excitation pulse is selected by a half-wave plate (HWP) and a quarter-wave plate (QWP). THz waves radiated from the sample are observed by a photoconductive antenna at a 45° angle. The polarization of THz wave is selected by two wire-grid polarizers (WGP). (**b**,**c**) S- and p-polarized THz waveforms measured under the s-, p-, R- and L-polarized excitations. X_Ex_ (X = S, P, R, and L) represents the polarization of the excitation pulse. Solid red lines are the fitted curves. (**d**) Schematic of optical setup of time-resolved magneto-optical Kerr rotation measurements. R- and L-polarized excitation pulses, and time-delayed, s-polarized probe pulses are directed at the sample at an incident angle of 45° and ~36°, respectively. The Kerr rotation signals are obtained by measuring the difference in signals between the reflected probe pulses through a HWP and a polarizing beam splitter (PBS) detected by two detectors (Det. 1 and Det. 2). (**e**,**f**) Time-resolved magneto-optical Kerr rotation signals under the R- and L-polarized excitations (solid black curves). Solid red curves are the fitted results from Eq. (3). Solid blue curves are the components relative to the spin relaxation. (**g**) Schematic illustration of photo-excitation process in Bi_2_Te_3_^27,28^. BVB, BCB and SS represent the bulk valence band, bulk conduction band and surface state, respectively. A red arrow indicates the transition from BVB_1_ to SS_2_.

In response to excitation pulses applied with various polarization (Fig. 1a), we observed THz waves radiated from photo-excited carriers in a fabricated Bi_2_Te_3_ TI thin film, which has polycrystals preferentially oriented with the *c*-axis perpendicular to the sample surface (see Supplementary Information). The s- and p-polarized THz waveforms from the Bi_2_Te_3_ thin film under the s-, p-, right-circularly (R) and left-circularly (L) polarized excitations are shown in Fig. 1b,c. We observed p-polarized THz waves in response to all the polarized excitations, while s-polarized THz waves were observed only in response to circularly polarized excitations. Further, we found that under the R- and L-polarized excitations the amplitudes of the s-polarized THz waves are inverted relative to one another. This indicates inversion of the flow direction of the photocurrents along the perpendicular direction (*x* direction) to the incident plane (*y-z* plane) at the surface of the Bi_2_Te_3_ thin film.

**Figure 2:**
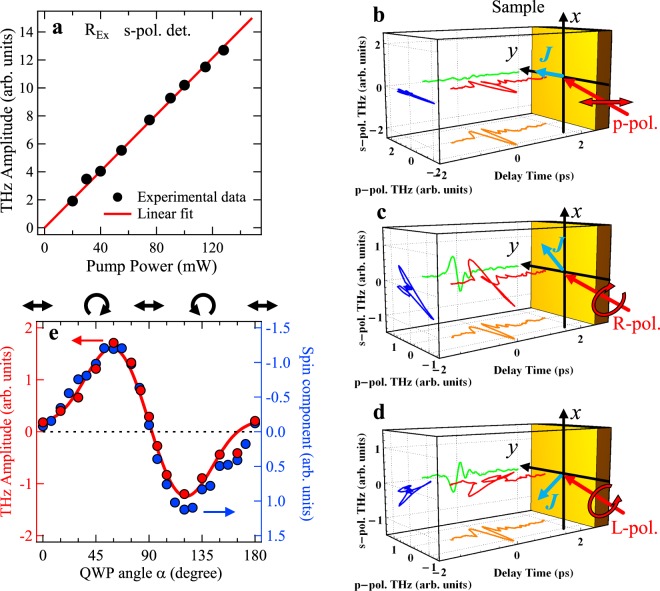
Control of flow directions of photocurrents generated in Bi_2_Te_3_ TI thin films by variable polarization of excitation pulses. (**a**) Excitation power dependence of amplitudes (dip-to-peak) of the s-polarized THz waves under the R-polarized excitation (black circles). The solid red line gives the linear fit result. (**b**–**d**) 3D plots of the THz waves under the p-, R- and L-polarized excitations using the s-polarized THz waveforms and the corrected p-polarized THz waveforms obtained by subtracting the raw p-polarized THz waveform under the s-polarized excitation from those under the p-, R-, and L-polarized excitations. Blue arrows represent the in-plane components of the photocurrents estimated from the calculation of the PGE. (**e**) Dependence of amplitudes (dip-to-peak) of s-polarized THz waves (red circles) and the spin component in Kerr rotation signals (blue circles) on rotation angle of a quarter-wave plate (QWP), *α*. As *α* varies, the polarization of the excitation pulse changes such as p-polarization (*α* = 0°), R-polarization (*α* = 45°), p-polarization (*α* = 90°), L-polarization (*α* = 135°), and p-polarization (*α* = 180°). The solid red curve is the fitted result to the *α*-dependence of s-polarized THz amplitudes with Eq. (2).

When photocurrents are excited at the surface of the semiconductors, the photo-Dember currents and the transient drift currents, which flow along the depth direction, generally radiate as p-polarized THz waves, not s-polarized THz waves^18,19^. Hence, in order to verify the origin of the s-polarized THz waves from the Bi_2_Te_3_ thin film, we measured the s-polarized THz waves by varying the excitation intensity under the R-polarized excitation. The excitation intensity dependence of the amplitude (dip-to-peak) of the s-polarized THz waveform shown in Fig. 2a clearly indicates that the amplitude depends linearly on excitation intensity, i.e. quadratic dependence on the electric field of the excitation pulse. This result means that the photocurrents at the surface of the Bi_2_Te_3_ thin film are generated by the photogalvanic effect (PGE), which is represented by^20^

1 $${J}_{\lambda }^{{\rm{PGE}}}={\sum }_{\mu ,\nu }{\sigma }_{\lambda \mu \nu }{E}_{\mu }{E}_{\nu }^{\ast }\,(\lambda ,\mu ,\nu =x,y,z).$$

Here, ${\sigma }_{\lambda \mu \nu }(={\sigma }_{\lambda \mu \nu }^{{\rm{Re}}}+i{\sigma }_{\lambda \mu \nu }^{{\rm{Im}}})$ is a third rank complex tensor called the photogalvanic tensor, and *E* represents the electric field of the excitation pulse. Applying the symmetry of the surface of Bi_2_Te_3_ (*C*_3*v*_) to ${\sigma }_{\lambda \mu \nu }$ and the polarization state of the excitation pulse to *E*, the PGE current ${J}_{\lambda }^{{\rm{PGE}}}$ can be calculated^21^ (Table 1), where the terms related to azimuthal angle are excluded in the calculation of the PGE currents since the fabricated Bi_2_Te_3_ thin film has a polycrystalline structure. Note that ${J}_{\lambda }^{{\rm{PGE}}}$ vanishes in the bulk of Bi_2_Te_3_ (${D}_{3d}^{5}$) due to symmetry considerations^22^. The inverted amplitude of the s-polarized THz wave between the R- and L- polarized excitations and the observed lack of s-polarized THz waves under the linearly polarized excitations shown in Fig. 1b are well accounted for by the calculated PGE current. This indicates that the THz waves emitted from the Bi_2_Te_3_ thin film originate from the photocurrent generated by the PGE.

**Table 1:** PGE current ${{J}}_{{\lambda }}^{PGE}$ calculated by taking the Fresnel coefficients into account.

Ex./Det.	*x*: S	*y*: P	*z*: P
S	0	0	${E}_{0}^{2}{|{t}_{s}|}^{2}{\sigma }_{zxx}^{{\rm{Re}}}$
P	0	${E}_{0}^{2}{|{t}_{p}|}^{2}{\sigma }_{yyz}^{{\rm{Re}}}\,\sin \,2\theta$	${E}_{0}^{2}{|{t}_{p}|}^{2}{\sigma }_{zxx}^{{\rm{Re}}}{\cos }^{2}\theta +{E}_{0}^{2}{|{t}_{p}|}^{2}{\sigma }_{zzz}^{{\rm{Re}}}{\sin }^{2}\theta$
R	${E}_{0}^{2}|{t}_{s}{t}_{p}|({\sigma }_{xxz}^{{\rm{Im}}}\,\cos \,\delta -{\sigma }_{xxz}^{{\rm{Re}}}\,\sin \,\delta )\,\sin \,\theta$	$\frac{1}{2}{E}_{0}^{2}{|{t}_{p}|}^{2}{\sigma }_{yyz}^{{\rm{Re}}}\,\sin \,2\theta$	$\frac{1}{2}{E}_{0}^{2}{\sigma }_{zxx}^{{\rm{Re}}}({|{t}_{s}|}^{2}+{|{t}_{p}|}^{2}{\cos }^{2}\theta )+\frac{1}{2}{E}_{0}^{2}{|{t}_{p}|}^{2}{{\rm{\sigma }}}_{zzz}^{{\rm{Re}}}\,{\sin }^{2}\theta$
L	$-{E}_{0}^{2}|{t}_{s}{t}_{p}|({\sigma }_{xxz}^{{\rm{Im}}}\,\cos \,\delta -{\sigma }_{xxz}^{{\rm{Re}}}\,\sin \,\delta )\,\sin \,\theta$	$\frac{1}{2}{E}_{0}^{2}{|{t}_{p}|}^{2}{\sigma }_{yyz}^{{\rm{Re}}}\,\sin \,2\theta$	$\frac{1}{2}{E}_{0}^{2}{\sigma }_{zxx}^{{\rm{Re}}}({|{t}_{s}|}^{2}+{|{t}_{p}|}^{2}{\cos }^{2}\theta )+\frac{1}{2}{E}_{0}^{2}{|{t}_{p}|}^{2}{\sigma }_{zzz}^{{\rm{Re}}}\,{\sin }^{2}\theta$

The first column indicates the polarization of the excitation pulse. The first row indicates the direction of the photocurrent (*x, y, z*) and the polarization of the THz waves (s,p). *E*_0_ and *θ* are the electric field and incident angle of the excitation pulse, respectively. *t*_s_ and *t*_p_ are the transmission Fresnel coefficients for the s- and p-polarized light, respectively, and *δ* is the phase retardation caused at the interface between the s- and p-polarized light. ${\sigma }_{\lambda \mu \nu }(={\sigma }_{\lambda \mu \nu }^{{\rm{Re}}}+i{\sigma }_{\lambda \mu \nu }^{{\rm{Im}}})$ is the photogalvanic tensor with the symmetry of the surface of Bi_2_Te_3_ (C_3*v*_). The real part ${\sigma }_{\lambda \mu \nu }^{{\rm{Re}}}$ has four independent elements given by ${\sigma }_{xxx}^{{\rm{Re}}}=-\,{\sigma }_{xyy}^{{\rm{Re}}}=-\,{\sigma }_{yyx}^{{\rm{Re}}}=-\,{\sigma }_{yxy}^{{\rm{Re}}}$, ${\sigma }_{xxz}^{{\rm{Re}}}={\sigma }_{xzx}^{{\rm{Re}}}={\sigma }_{yyz}^{{\rm{Re}}}={\sigma }_{yzy}^{{\rm{Re}}}$, ${\sigma }_{zxx}^{{\rm{Re}}}={\sigma }_{zyy}^{{\rm{Re}}}$, ${\sigma }_{zzz}^{{\rm{Re}}}$. The other elements of the real part vanish. The imaginary part ${\sigma }_{\lambda \mu \nu }^{{\rm{Im}}}$ has only one element ${\sigma }_{xxz}^{{\rm{Im}}}=-\,{\sigma }_{xzx}^{{\rm{Im}}}={\sigma }_{yyz}^{{\rm{Im}}}=-\,{\sigma }_{yzy}^{{\rm{Im}}}$.

In order to determine the flow direction of the photocurrent in the Bi_2_Te_3_ thin film, we perform 3D plots of the THz waveforms using the s- and p-polarized THz waves. However, we experience a difficulty in making the 3D plots from the p-polarized THz waveforms including the ${\sigma }_{zxx}^{{\rm{Re}}}$ term because the p-polarized THz waveform under the s-polarized excitation shows a phase shift as compared with the other p-polarized THz waveforms. Therefore, we obtained corrected p-polarized THz waveforms (Fig. S4 in Supplementary Information) by subtracting the raw p-polarized THz waveform under the s-polarized excitation from those under the p-, R-, and L-polarized excitations. The 3D plots of the THz waveforms under the p-, R- and L-polarized excitations depicted in Fig. 2b–d indicate that the THz wave radiated from the PGE currents flowing in the Bi_2_Te_3_ thin film is projected to the *x-y* plane. It is clear that the polarization of the THz wave depends on the polarization of the excitation pulse. The THz wave under the p-polarized excitation exhibits linear polarization along the *y* direction, which indicates that the PGE current flows in the *y*-*z* plane. Under the R-polarized excitation, the THz wave exhibits almost linear polarization inclining at about 45° from the *y* axis, while the polarization direction of the THz wave under the L-polarized excitation is inclined at about −45°. These results demonstrate that the flow direction of the photocurrent changes with the polarization of the excitation pulse.

We expect that the flow direction of the photocurrent will depend on the ellipticity of the excitation pulse polarization. To investigate this dependence, we measured the s-polarized THz waves by rotating a quarter-wave plate (QWP) through an angle *α*, which changes the ellipticity of the excitation pulse polarization. The *α*-dependence of the s-polarized THz wave amplitude in Fig. 2e indicates that the magnitude of the photocurrents flowing in the *x* direction at the surface of the sample continuously changes with the ellipticity of the polarization of the excitation pulse, while background photocurrent due to the Seebeck effect is hardly observed. In order to verify the ellipticity dependence of the photocurrent, we calculate the photocurrent flowing in the *x* direction due to the PGE. The *x* component of the photocurrent, which is dependent on *α*, is represented by (see Supplementary Information)

2 $$\begin{array}{lll}{J}_{x}^{{\rm{PGE}}} & \propto  & C\,\sin \,2\alpha +L\,\sin \,4\alpha +D,\\ C & = & {\sigma }_{xxz}^{{\rm{Im}}}\,\cos \,\delta -{\sigma }_{yyz}^{{\rm{Re}}}\,\sin \,\delta ,\\ L & = & -\,\frac{1}{2}[{\sigma }_{xxz}^{{\rm{Im}}}\,\sin \,\delta +{\sigma }_{yyz}^{{\rm{Re}}}\,\cos \,\delta ],\end{array}$$

where *δ* is the phase retardation appearing at the interface between the s- and p-polarized light (*δ* is estimated to be approximately 3.5° from the Fresnel coefficients of *t*_s_ and *t*_p_)^23^, and D indicates the bulk thermoelectric current due to the Seebeck effect. The *α*-dependence of the s-polarized THz wave amplitude is fitted with Eq. (2) as indicated by the red curve in Fig. 2e, which is in good agreement with the experimental result. This agreement illustrates that excitation pulses with various polarization ellipticities enable one to control the direction of the photocurrent generated at the surface of the Bi_2_Te_3_ thin film while suppressing bulk thermoelectric currents (C:L:D = 1:0.8:0.15).

### Kerr rotation measurements

Since Dirac electrons in the TI thin film exhibit spin-momentum locking, the spin polarization of surface Dirac electrons is oriented in the *x*-*y* plane, perpendicular to the direction of the photocurrent^1–3^. Hence we expect that the spin polarizations of the photocurrents under the R- and L-polarized excitations will be inverted relative to one another. Therefore, we have investigated the spin polarization under circular polarized excitations by measuring the time-resolved magneto-optical Kerr rotation (Fig. 1d). The R- and L-polarized pump pulses were focused on the Bi_2_Te_3_ thin film at an incident angle of 45° in the same way as the previous THz wave measurements. The s-polarized probe pulses were focused on the sample at an incident angle of ~36° since the Kerr rotation angle becomes 0 for normal incidence (see Supplementary Information). The Kerr rotation signals under the R- and L-polarized excitations are shown in Fig. 1e,f, respectively. Sign inversion of the Kerr rotation signals is clearly observed between the two excitations. To confirm the time evolution of the Kerr rotation signals *ξ*_*K*_, the experimental data are fitted in Fig. 1e,f (solid red curve) with^15,24^

3 $$\begin{array}{rcl}{\xi }_{K}(t) & = & {A}_{1}\,\exp (-\frac{{t}^{2}}{{\sigma }^{2}})+{A}_{2}\,\exp (-\frac{t}{{\tau }_{S}}+\frac{{\sigma }^{2}}{4{\tau }_{S}^{2}})\times [1+{\rm{erf}}(\frac{t}{\sigma }-\frac{\sigma }{2{\tau }_{S}})]\\  &  & +\,{A}_{3}\,\exp (-\frac{t}{{\tau }_{BC}}+\frac{{\sigma }^{2}}{4{\tau }_{BC}^{2}})\times [1+{\rm{erf}}(\frac{t}{\sigma }-\frac{\sigma }{2{\tau }_{BC}})],\end{array}$$

where *σ* is the excitation pulse width, and *τ*_S_ is the spin relaxation time. The first and second terms describe instantaneous spin contributions of the bulk carriers^25,26^ and non-instantaneous spin contributions of the surface carriers, respectively. Note that the *y* and *z* components of the spin-polarization are observed in the present optical configuration. Consequently, for the surface carriers only the *y* component of the spin polarization is observed, while for the bulk carriers the *y* and *z* components of the spin polarization are both observed because the spin polarizations of the bulk electrons are oriented in various directions. The third term represents the bulk carrier contributions with the relaxation time of τ_BC_, which behave as background signals, because the amplitude A_3_ has negligibly small values and τ_BC_ is estimated to be ~2 ps. The components due to the spin relaxation related to the second term in Eq. (3) are marked with solid blue curves in Fig. 1e,f. The Kerr rotation signals corresponding to the spin dynamics are obviously reversed between the R- and L-polarized excitations, which indicates inversion of the spin polarization of the photo-excited carriers. Moreover, in order to verify the relationship between spin polarization and photocurrent, we investigated the *α*-dependence of the Kerr rotation signals (blue circles in Fig. 2e). The *α*-dependence of the spin component is on the order of THz amplitude. This result indicates that the degree of spin polarization in the *y* direction depends on the flow direction of the photocurrent (see Supplementary Information). Consequently, it is clear that the flow direction of the spin-polarized photocurrents can be controlled by the polarization of the excitation pulse alone.

## Discussion

Finally, we discuss the relaxation process of the spin-polarized photocurrent. The spin relaxation time is estimated to be *τ*_S_ ~ 15 fs from the above fitting procedure for the Kerr rotation signals. On the other hand, the relaxation time of the photo-excited carriers is estimated to be *τ*_SC_ ~ 25 fs by fitting the data in Fig. 1b with a function obtained from the convolution of the relaxation profile of the photo-excited carriers and the Gaussian pulse profile (see Supplementary Information). We notice that *τ*_SC_ is the same order as the spin relaxation time. From the electronic band structure of Bi_2_Te_3_ TIs shown in Fig. 1g^27,28^, the excitation pulses with center energy of 1.55 eV induce the optical transition from the bulk valence band (BVB_1_) to the unoccupied second surface state (SS_2_) at higher energy compared to the first surface state (SS_1_). Hence the observed THz waves are radiated from the photocurrent due to the photo-excited carriers in SS_2_. The estimated relaxation time of the photocurrent *τ*_SC_ is much shorter than the relaxation time due to electron-phonon scattering (500 fs)^29^ and the scattering time (300–450 fs) at the grain boundary that is calculated from the Fermi velocity of Bi_2_Te_3_ (~3.3 × 10^5^ m/s)^30^ and grain size (0.2–0.3 μm). Besides scattering due to defects, impurities and carriers at the surface, therefore we suggest that the relaxation time of the photo-excited carriers *τ*_SC_ will originate from the escape of photo-excited carriers to the bulk because the flow direction of the photocurrent includes the *z* component relative to the depth direction. Moreover, when the spin-polarized photocurrent is transported from the surface to the bulk, the spin polarization will be immediately depolarized since the bulk states in Bi_2_Te_3_ do not experience spin-momentum locking. Next we consider that the obtained spin relaxation time *τ*_S_ is attributable to the spin relaxation of the photo-excited carriers in SS_2_. The results obtained in the present study indicate that the spin-polarized photo-excited carriers generated in SS_2_ exhibit a high-speed response due to the fast relaxation from SS_2_ to the second bulk conduction band (BCB_2_).

In conclusion, we have demonstrated that excitation pulses with various polarizations can be used to precisely and intentionally control the flow direction of spin-polarized photocurrents generated in a Bi_2_Te_3_ TI thin film via the photogalvanic effect. Our results could likely play an important role in the development of optoelectronic and spintronic TI devices.

## Electronic supplementary material

Supplementary Information
