# Supplementary material for: Optical control of spin-polarized photocurrent in topological insulator thin films
Source: Sci Rep. 2018 Oct 18;8:15392. doi: 10.1038/s41598-018-33716-0 (PMC6193961; doi:10.1038/s41598-018-33716-0)
Supplement: Supplementary file 1 — Supplementary Information [file 41598_2018_33716_MOESM1_ESM.docx]

Supplementary Information for Optical control of spin-polarized photocurrent in topological insulator thin films

H. Takeno, S. Saito, and K. Mizoguchi

1. Crystal structure of Bi_2_Te_3_

The crystal structure of Bi_2_Te_3_ used in experiments is shown in Fig. S1. Bi_2_Te_3_ has a layered structure consisting of a quintuple layer (Te-Bi-Te-Bi-Te) stacked along the c-axis. The crystal structure of Bi_2_Te_3_ belongs to the space group $D_{3d}^{5}$, while the surface of Bi_2_Te_3_, which is the topological layer, has $C_{3v}$ symmetry^1,2^.

1. Sample fabrication, X-ray diffraction measurement and AFM measurement

The Bi_2_Te_3_ thin film samples were grown to a thickness of 15 nm on (0001)-Al_2_O_3_ substrates by using a vacuum deposition method. An X-ray diffraction pattern of the fabricated sample is shown in Fig. S2(a) in which the (0 0 3n) Miller indices represent the layered structure of Bi_2_Te_3_ along the c-axis. The diffraction pattern confirms that the fabricated Bi_2_Te_3_ thin film has a layered structure stacked along the c-axis, with diffraction peaks corresponding only to the (0 0 3n) Miller indices. An AFM image of the surface of the Bi_2_Te_3_ thin film (10×10 μm^2^) is shown in Fig. S2(b), which indicates that the sample is composed of grains with diameter of 0.2-0.3 μm that are preferentially oriented along the *c*-axis, perpendicular to the sample surface.

1. Polarized Raman measurement

We performed the polarized Raman measurements at room temperature using a Raman spectroscopy system (LabRAM HR). We used a cw laser with a center wavelength of 532 nm as the excitation source for Raman scattering, which was focused on the sample by an objective lens with a numerical aperture of 0.75. The spot size was about 1 μm. The polarized Raman spectra of the Bi_2_Te_3_ thin film were measured in the back scattering configuration of $Z(X, X)\bar{Z}$ by rotating the sample through the azimuthal angle **, as shown in Fig. S3(a). The Raman spectrum at an azimuthal angle of ** = 0º is shown in Fig. S3(b). Two phonon modes are observed at 102 and 134 cm^-1^, which are attributed to the E_g_ and A_1g_ modes of Bi_2_Te_3_, respectively^3^. The observed Raman spectra at various azimuthal angles were fitted by two superposed Lorentz functions. The fitted curve is marked in red in Fig. S3(b). The azimuthal angle dependence of the intensity ratio of the E_g_ mode to the A_1g_ mode (E_g_/A_1g_) is shown in Fig. S3(c). This result indicates that the intensity ratio of two modes E_g_/A_1g_ is independent of the azimuthal angle. Here, we consider the dependence of the Raman intensities of the E_g_ and A_1g_ modes in a Bi_2_Te_3_ single crystal on the polarization angle * *of the incident and scattered light, which is calculated from the Raman tensors shown in Table S1. The intensity of the A_1g_ mode is independent of **, while the intensity of the E_g_ mode is proportional to |sin2**|^2^. Therefore, when the Bi_2_Te_3_ thin film has a single crystal structure, the intensity ratio of the two modes E_g_/A_1g_ should exhibit |sin2**|^2^ dependence. However, this contradicts our observation that the azimuthal dependence is constant. This discrepancy can be explained as follows: The intensity of the E_g_ mode is effectively averaged over ** because the Bi_2_Te_3_ thin film sample has a polycrystalline structure consisting of many grains that are randomly oriented in-plane. Hence, the results of X-ray diffraction measurement, AFM measurement and polarized Raman spectroscopy consistently indicate that the fabricated Bi_2_Te_3_ thin film is composed of grains with diameter of 0.2-0.3 μm that are preferentially oriented with the *c*-axis perpendicular to the sample surface, while the crystal axes in the surface plane of the grains are randomly oriented.

1. THz wave measurement

The THz wave measurements have been performed at room temperature in a box filled with dry N_2_ gas, in order to avoid the absorption of THz waves due to water vaper. The THz waves radiated from photo-excited carriers in the Bi_2_Te_3_ thin film have been observed by irradiating the excitation pulses with various polarizations (Fig. 1a in the main text). The excitation pulses with s-, p-, R- and L-polarizations from a Ti:sapphire pulse laser (center wavelength:800 nm (1.55 eV), pulse width:~60 fs, repetition rate:80 MHz, spot size:~30µm) were focused on the sample at an incident angle of 45°. The polarization of the excitation pulses was selected by a half-wave plate (HWP) and a quarter-wave plate (QWP). The s- and p-polarized THz waves through the wire-grid polarizers were detected by a photoconductive antenna.

1. Fitting function to the time-domain THz waveform

To estimate the relaxation time of the photo-excited carriers, we fit the s-polarized THz waveforms under the R- and L-polarized excitations with the following equation. This equation is obtained from the convolution of the relaxation profile of the photo-excited carriers and the Gaussian pulse profile,

|  | $E_{\mathrm{THz}}\left( t \right)\propto\frac{dJ\left( t \right)}{dt}\propto\frac{d}{dt}\int_{-\infty}^{\infty} \Theta\left( t^{'} \right) e^{- \frac{t^{'}}{\tau_{SC}}} e^{- \left( \frac{t^{'}-t}{\sigma} \right)^{2}}dt^{'}\propto\frac{d}{dt}\left[ \left( \mathrm{erf}\left( t/\sigma\right)+1 \right)e^{- \frac{t}{\tau_{SC}}} \right]$ , | (S1) |
| --- | --- | --- |

where $\Theta$ is the Heaviside step function, erf is the error function, *σ* is the pulse width of the excitation pulse and *τ*_SC_ is the relaxation time of the photo-excited surface carriers.

1. Calculation of photogalvanic effect (PGE) current

The photogalvanic effect (PGE) current *J*^PGE^ is described by

|  | $J_{\lambda}^{\mathrm{PGE}}=\sum_{\mu, \nu} \sigma_{\lambda\mu\nu}E_{\mu}E_{\nu}^{*} \left( \lambda, \mu, \nu=x, y, z \right)$ . | (S2) |
| --- | --- | --- |

Here, $\sigma_{\lambda\mu\nu}\left( =\sigma_{\lambda\mu\nu}^{\text{Re}}+i\sigma_{\lambda\mu\nu}^{\text{Im}} \right)$ is the photogalvanic tensor and $E_{\mu}$($E_{\nu}$) represents the electric field of the excitation pulse. Using the polar coordinate system shown in Fig. 1a in the main text (incident angle:* *, azimuthal angle: **), after the p-polarized pulse is passed through a quarter-wave plate (QWP) the electric field of the excitation pulse $\boldsymbol{E}=\left( E_{x}, E_{y}, E_{z} \right)$ is given by

|  | $\boldsymbol{E}\propto\left( \begin{matrix} -it_{s}\sin2\alpha\cos\varphi+t_{p}\left( 1+i\cos2\alpha\right)\cos\theta\sin\varphi\\ i t_{s}\sin2\alpha\sin\varphi+t_{p}\left( 1+i\cos2\alpha\right)\cos\theta\cos\varphi\\ t_{p}\left( 1+i\cos2\alpha\right)\sin\theta\end{matrix} \right)$ . | (S3) |
| --- | --- | --- |

Here, *t*_s_ and *t*_p_ are the transmission Fresnel coefficients for the s- and p-polarized light, respectively, while *α* is the rotation angle of the QWP. The ellipticity of the excitation pulse polarization changes with *α*, such as p-polarization (*α*=0°), R-polarization (*α*=45°), p-polarization (*α*=90°), L-polarization (*α*=135°), and p-polarization (*α*=180°). By substituting Eq. (S3) and the photogalvanic tensor with the surface symmetry of Bi_2_Te_3_ (C_3_*_v_*) into Eq. (S2), the PGE current is calculated as follows,

|  | $\boldsymbol{J}^{\mathrm{PGE}}={\frac{1}{2}E}_{0}^{2}\left\vert t_{s}t_{p} \right\vert\sigma_{xxz}^{\mathrm{Im}}\left( 2\sin2\alpha\cos\delta-\sin4\alpha\sin\delta\right)\sin\theta\left( \begin{matrix} 1 \\ 0 \\ 0 \end{matrix} \right)$ $-\frac{1}{4}E_{0}^{2}\sigma_{xxx}^{\mathrm{Re}}\left[ 2\left\vert t_{s}t_{p} \right\vert\left( \sin4\alpha\cos\delta+2\sin2\alpha\sin\delta\right)\cos\theta\left( \begin{matrix} \sin3\varphi\\ -\cos3\varphi\\ 0 \end{matrix} \right)-\left\{ \left\vert t_{s} \right\vert^{2}\left( 1-\cos4\alpha\right)-\left\vert t_{p} \right\vert^{2}\left( 3+\cos4\alpha\right)\cos^{2} \theta\right\}\left( \begin{matrix} \cos3\varphi\\ \sin3\varphi\\ 0 \end{matrix} \right) \right]$ $+\frac{1}{4}E_{0}^{2}\sigma_{yyz}^{\mathrm{Re}}\left[ -2\left\vert t_{s}t_{p} \right\vert\left( \sin4\alpha\cos\delta+2\sin2\alpha\sin\delta\right)\sin\theta\left( \begin{matrix} 1 \\ 0 \\ 0 \end{matrix} \right)+\left\vert t_{p} \right\vert^{2}\left( \cos4\alpha+3 \right)\sin2\theta\left( \begin{matrix} 0 \\ 1 \\ 0 \end{matrix} \right) \right]$ $+\frac{1}{4}E_{0}^{2}\sigma_{zxx}^{\mathrm{Re}}\left[ \left\vert t_{s} \right\vert^{2}\left( 1-\cos4\alpha\right)+\left\vert t_{p} \right\vert^{2}\left( 3+\cos4\alpha\right)\cos^{2} \theta\right]\left( \begin{matrix} 0 \\ 0 \\ 1 \end{matrix} \right)+\frac{1}{4}E_{0}^{2}\left\vert t_{p} \right\vert^{2}\sigma_{zzz}^{\mathrm{Re}}\left( \cos4\alpha+3 \right)\sin^{2} \theta\left( \begin{matrix} 0 \\ 0 \\ 1 \end{matrix} \right) .$ | (S4) |
| --- | --- | --- |

Here, *δ* is the phase retardation appearing at the interface between the s- and p-polarized light, expressed by $t_{s}/t_{p}=\left| t_{s}/t_{p} \right|e^{-i\delta}$. (100), (010), and (001) are the unit vectors along the *x* axis, *y* axis and *z* axis, respectively. The *x*, *y*, and *z* components of the calculated PGE current, which correspond to the polarized THz waves, are listed in Table 1 in the main text.

The general expression for the PGE currents include the *z* components describing the photocurrent flowing in the depth direction. The *z* component under the s-polarized excitation is represented only by the term $\sigma_{zxx}^{\mathrm{Re}}$. The components of the PGE currents remaining after eliminating the $\sigma_{zxx}^{\mathrm{Re}}$ term are listed in Table S2. Moreover, the corrected p-polarized THz waveforms by subtracting the raw p-polarized THz waveform under the s-polarized excitation from those under the p-, R-, and L-polarized excitations are shown in Fig. S4(c). The amplitude (dip-to-peak) of the corrected p-polarized THz waveform under the p-polarized excitation is about two times larger than those under the R- and L-polarized excitations, which is consistent with that obtained by the calculated PGE currents shown in Table S2.

1. Time-resolved magneto-optical Kerr rotation measurement

The time-resolved magneto-optical Kerr rotation measurements were performed using the reflection-type pump-probe method at room temperature (Fig. 1d in the main text). The excitation pulses with R- and L-polarizations from a Ti:sapphire pulse laser (center wavelength:800 nm (1.55 eV), pulse width:~60 fs, repetition rate:80 MHz, spot size:~30µm) were focused on the sample at an incident angle of 45°. The time-delayed probe pulses with s-polarization were directed at the sample at an incident angle of ~36°. The reflected probe pulses through a HWP and a polarizing beam splitter (PBS) were measured by using balance detection with two detectors (Det. 1 and Det. 2). The Kerr rotation signals are expressed as the difference between signals from the reflected probe pulses detected by two detectors.

1. Fitting to the time-resolved magneto-optical Kerr rotation signals

We measured the Kerr rotation signals at various QWP angle of *α* (black curves in Fig. S5). One can see that these signals change with *α*. We fit all the Kerr rotation signals with Eq. (3) in the main text. All fitted results (red curves in Fig. S5) reproduce well the experimental results.

1. *α*-dependence of the spin component in Kerr rotation signals

Dirac electrons at the topological surface state exhibit the characteristic of the spin-momentum locking. Therefore, the photocurrent dependent on the polarization of the excitation pulses should have the spin polarization oriented with the direction perpendicular to the photocurrent. The degree of spin polarization can be investigated by the magneto-optical Kerr rotation measurement. Here we calculate the relationship between the Kerr rotation signal due to the spin polarization in the *y* direction **_K_ and the photocurrent in the *x* direction *J_x_*. When spin polarization due to the photo-excited carriers is oriented with the *y* direction, the magnetization *M_y_* is generated in the same direction,

|  | $M_{y}=N_{x}\left\langle\mu_{y} \right\rangle$ . | (S5) |
| --- | --- | --- |

Here, *N_x_* is the density of the photo-excited carriers with the momentum in the *x* direction and $\left\langle\mu_{y} \right\rangle$ is the average of the magnetic moment in the *y* direction, which is attributed to the spin polarization. The dielectric tensor of the magnetized sample is represented by^4^

|  | $\varepsilon(M_{y})=\left( \begin{matrix} \varepsilon_{xx}(M_{y}) & 0 & \varepsilon_{xz}(M_{y}) \\ 0 & \varepsilon_{xx}(M_{y}) & 0 \\ -\varepsilon_{xz}(M_{y}) & 0 & \varepsilon_{xx}(M_{y}) \end{matrix} \right)$ . | (S6) |
| --- | --- | --- |

Under the assumption that the effect of the magnetization *M_y_* on the dielectric tensor is very small, $\varepsilon_{ij}(M_{y})$ can be expanded as a power series of *M_y_* ^5^,

|  | $\varepsilon_{ij}\left( M_{y} \right)=\sum_{n=0, 1, 2,\cdots} {\varepsilon_{ij}}^{(n)}{M_{y}}^{n}$ , | (S7) |
| --- | --- | --- |

where ${\varepsilon_{ij}}^{(n)}$ is the coefficients independent of *M_y_*. $\varepsilon_{ij}\left( M_{y} \right)$ obeys the Onsager relation^6^

|  | $\varepsilon_{ij}\left( M_{y} \right)=\varepsilon_{ji}\left( -M_{y} \right)$ . | (S8) |
| --- | --- | --- |

Substituting Eq. (S7) and Eq. (S8) into Eq. (S6), $\varepsilon(M_{y})$ is rewritten as

|  | $\varepsilon(M_{y})=\left( \begin{matrix} {\varepsilon_{xx}}^{(0)} & 0 & {\varepsilon_{xz}}^{(1)}M_{y} \\ 0 & {\varepsilon_{xx}}^{(0)} & 0 \\ -{\varepsilon_{xz}}^{(1)}M_{y} & 0 & {\varepsilon_{xx}}^{(0)} \end{matrix} \right)$ , | (S9) |
| --- | --- | --- |

where the second and higher order terms in *M_y_* are neglected. When the polarized probe pulse is focused on the magnetized sample, the relation between the incident and reflected electric fields of the probe pulses is expressed as

|  | $\left( \begin{matrix} E_{s}^{r} \\ E_{p}^{r} \end{matrix} \right)=\left( \begin{matrix} r_{ss} & r_{sp} \\ r_{ps} & r_{pp} \end{matrix} \right)\left( \begin{matrix} E_{s}^{i} \\ E_{p}^{i} \end{matrix} \right)$ , | (S10) |
| --- | --- | --- |

where *E* is the electric field of the probe pulse, superscripts *i* and *r* denote the incident and reflected probe pulses, the subscripts *s* and *p* denote the polarization, and *r_mn_* (*m*, *n* = s, p) is the reflection Fresnel coefficient. When the s-polarized probe pulse is obliquely incident in *y*-*z* plane, $r_{ss}$ and $r_{ps}$ are written as^7,8^

|  | $r_{ss}=\frac{\cos\theta^{'}-\sqrt{{\varepsilon_{xx}}^{(0)}-\sin^{2} \theta^{'}}}{\cos\theta^{'}+\sqrt{{\varepsilon_{xx}}^{(0)}-\sin^{2} \theta^{'}}}$ , | (S11) |
| --- | --- | --- |

|  | $r_{ps}=\frac{\sin\theta^{'}\cos\theta^{'}{\varepsilon_{xz}}^{(1)}M_{y}}{\left( \cos\theta^{'}+\sqrt{{\varepsilon_{xx}}^{(0)}-\sin^{2} \theta^{'}} \right)\left( {\varepsilon_{xx}}^{(0)}\cos\theta^{'}+\sqrt{{\varepsilon_{xx}}^{(0)}-\sin^{2} \theta^{'}} \right)\sqrt{{\varepsilon_{xx}}^{(0)}-\sin^{2} \theta^{'}}}$ , | (S12) |
| --- | --- | --- |

where *'* is an incident angle of the probe pulse. When the Kerr rotation angle *θ*_K_ is assumed to take the small value, we obtain^7,9^

|  | $\theta_{K}=Re\left[ \frac{r_{ps}}{r_{ss}} \right]=\frac{\sin\theta'\cos\theta'{\varepsilon_{xz}}^{\left( 1 \right)}M_{y}}{\left( \cos\theta'-\sqrt{{\varepsilon_{xx}}^{\left( 0 \right)}-\sin^{2} \theta'} \right)\left( {\varepsilon_{xx}}^{\left( 0 \right)}\cos\theta'+\sqrt{{\varepsilon_{xx}}^{\left( 0 \right)}-\sin^{2} \theta'} \right)\sqrt{{\varepsilon_{xx}}^{\left( 0 \right)}-\sin^{2} \theta'}}\propto M_{y}$ . | (S13) |
| --- | --- | --- |

Note that *θ*_K_ becomes 0 for the normal incidence of the probe pulse (*’*=0). According to the Kerr effect, the electric field of reflected probe pulse changes from the linear polarization to the elliptical polarization with ellipticity *η_K_*(=Im[*r_ps_/r_ss_*]), and the major axis of the elliptical polarization is rotated by *θ*_K_. The electric field of the reflected probe pulse with the major axis rotated by *θ*_K_ and ellipticity *η_K_* is represented by

|  | $\left( \begin{matrix} E_{s}^{r} \\ E_{p}^{r} \end{matrix} \right)=\left( \begin{matrix} \sin\theta_{K}\cos\eta_{K}+i\cos\theta_{K}\sin\eta_{K} \\ \cos\theta_{K}\cos\eta_{K}-i\sin\theta_{K}\sin\eta_{K} \end{matrix} \right)$ . | (S14) |
| --- | --- | --- |

We discuss the observed Kerr rotation signals through a HWP by using the balance detection. The electric field of the probe pulse through the HWP is written as

|  | $\left( \begin{matrix} E_{s}^{r}' \\ E_{p}^{r}' \end{matrix} \right)=\frac{1}{\sqrt{2}}\left( \begin{matrix} \left( \cos\theta_{K}-\sin\theta_{K} \right)\cos\eta_{K}-i\left( \cos\theta_{K}+\sin\theta_{K} \right)\sin\eta_{K} \\ \left( \cos\theta_{K}+\sin\theta_{K} \right)\cos\eta_{K}+i\left( \cos\theta_{K}-\sin\theta_{K} \right)\sin\eta_{K} \end{matrix} \right)$ . | (S15) |
| --- | --- | --- |

The signals *I*_1_ and *I*_2_ measured by two detectors (Det. 1 and Det. 2 in Fig. 1d in the main text) are represented by

|  | $I_{1}=\left\vert E_{p}^{r}' \right\vert^{2}=\frac{1}{2}\left( 1+\sin{2\theta}_{K}\cos{2\eta}_{K} \right)$ ,  $I_{2}=\left\vert E_{s}^{r}' \right\vert^{2}=\frac{1}{2}\left( 1-\sin{2\theta}_{K}\cos{2\eta}_{K} \right)$ . | (S16) |
| --- | --- | --- |

The Kerr rotation signal *_K_* obtained by the balance detection is

|  | $\xi_{K}=\frac{I_{1}-I_{2}}{I_{1}+I_{2}}=\sin{2\theta}_{K}\cos{2\eta}_{K}\approx{2\theta}_{K}$ , | (S17) |
| --- | --- | --- |

where the Kerr rotation angle *θ*_K_ and ellipticity *η_K_* are assumed to take the small value. From Eq. (S5) the magnetization *M_y_* is proportional to *N_x_,* which depends on the magnitude of the PGE current in the *x* direction $J_{x}^{\text{PGE}}$. Consequently, the following relationship is obtained

|  | $\xi_{K}\propto\theta_{K}\propto M_{y}\propto J_{x}^{\text{PGE}}$ . | (S18) |
| --- | --- | --- |

This relationship means that the α-dependence of the spin component in Kerr rotation signals is related to that of the amplitudes of s-polarized THz waves as shown in Fig. 2e in the main text. Note that the values of the vertical axis in Fig. 1e and 1f (the main text) represent the Kerr rotation angle.

References

1. Larson, P. & Lambrecht, W. R. L. Electronic structure and magnetism in Bi_2_Te_3_, Bi_2_Se_3_, and Sb_2_Te_3_ doped with transition metals (Ti–Zn). *Phys. Rev. B* **78,** 195207 (2008).

2. Fu, L. Hexagonal warping effects in the surface states of the topological insulator Bi_2_Te_3_. *Phys. Rev. Lett.* **103,** 266801 (2009).

3. Kullmann, W. *et al.* Effect of Hydrostatic and Uniaxial Pressure on Structural Properties and Raman Active Lattice Vibrations in Bi_2_Te_3_. *Phys. Stat. Sol. (b)* **125,** 131–138 (1984).

4. Hunt, R. P. Magneto-optic scattering from thin solid films. *J. Appl. Phys.* **38,** 1652–1671 (1967).

5. Wettling, W. Magneto-optics of ferrites. *J. Magn. Magn. Mater.* **3,** 147–160 (1976).

6. Landau, L. D. & Lifshitz, E. M. *Electrodymanics of continuous media*. **8,** (Pergamon, 1960).

7. You, C.-Y. & Shin, S.-C. Generalized analytic formulae for magneto-optical Kerr effects. *J. Appl. Phys.* **84,** 541–546 (1998).

8. Yang, Z. J. & Scheinfein, M. R. Combined three-axis surface magneto-optical Kerr effects in the study of surface and ultrathin-film magnetism. *J. Appl. Phys.* **74,** 6810–6823 (1993).

9. Antonov, V., Harmon, B. & Yaresko, A. *Electronic structure and magneto-optical properties of solids*. (Kluwer Academic Publishers, 2004).

10. Flock, J., Dekorsy, T. & Misochko, O. V. Coherent lattice dynamics of the topological insulator Bi_2_Te_3_ probed by ultrafast spectroscopy. *Appl. Phys. Lett.* **105,** 011902 (2014).

Figure S1. (a) Schematic of Bi_2_Te_3_ crystal structure. (b) Top view of Bi_2_Te_3_ crystal structure from the *c*-axis direction.

(a)

(b)

Figure S2. (a) X-ray diffraction pattern of the fabricated Bi_2_Te_3_ thin film. (b) AFM image of the surface of the fabricated Bi_2_Te_3_ thin film (image size is 10×10 μm^2^).


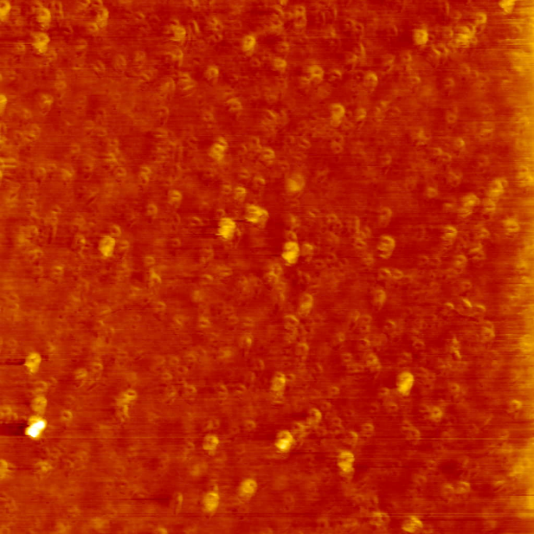


(a)

(b)

Figure S3. (a) Schematic of optical configuration of polarized Raman measurements. The polarization of the incident laser is parallel to that of the scattered light, which is detected in the backscattering configuration. (b) Raman spectrum of the Bi_2_Te_3_ thin film (solid black curve) and the fitted result with two superposed Lorentz functions (solid red curve). Two phonon modes at 102 cm^-1^ and 134 cm^-1^ correspond to the E_g_ and A_1g_ modes of Bi_2_Te_3_, respectively. (c) Azimuthal angle dependence of the Raman intensity ratio of E_g_ and A_1g_ modes (E_g_/A_1g_).


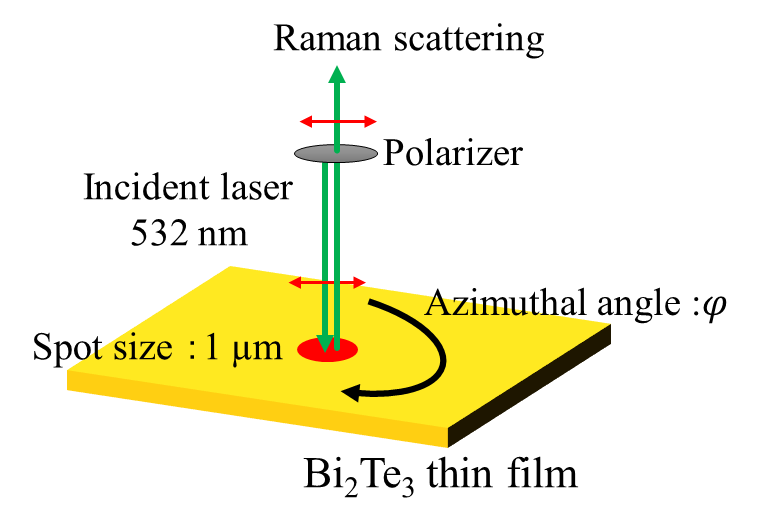


(a)

(b)

(c)

Figure S4. (a),(b) The s- and p-polarized THz waveforms under s-, p-, R- and L-polarized excitations, which are the same as those in Fig. 1b, c in the main text. (c) The corrected p-polarized THz waveforms obtained by subtracting the raw p-polarized THz waveform under the s-polarized excitation from those under the p-, R-, and L-polarized excitations.

(b)

(a)

(c)

Figure S5. The time-resolved magneto-optical Kerr rotation signals measured at various QWP angle of *α* (black curves) and the fitted results from Eq. (3) in the main text (red curves). All the fitted results reproduce well the experimental results.

Table S1. Raman tensors and polarization dependence of Raman intensities for A_1g_ and E_g_ modes in the Bi_2_Te_3_ crystal.

| Phonon mode | Raman Tensor^10^ | Polarization Dependence |
| --- | --- | --- |
| A_1g_ | $\left( \begin{matrix} a & 0 & 0 \\ 0 & a & 0 \\ 0 & 0 & a \end{matrix} \right)$ | $\left\vert\left( \begin{matrix} \sin\varphi& \cos\varphi& 0 \end{matrix} \right)\left( \begin{matrix} a & 0 & 0 \\ 0 & a & 0 \\ 0 & 0 & a \end{matrix} \right)\left( \begin{matrix} \sin\varphi\\ \cos\varphi\\ 0 \end{matrix} \right) \right\vert^{2}=a^{2}$ |
| E_g_ | $\left( \begin{matrix} 0 & c & 0 \\ c & 0 & d \\ 0 & d & 0 \end{matrix} \right)$ | $\left\vert\left( \begin{matrix} \sin\varphi& \cos\varphi& 0 \end{matrix} \right)\left( \begin{matrix} 0 & c & 0 \\ c & 0 & d \\ 0 & d & 0 \end{matrix} \right)\left( \begin{matrix} \sin\varphi\\ \cos\varphi\\ 0 \end{matrix} \right) \right\vert^{2}= \left\vert c\cdot\sin2\varphi\right\vert^{2}$ |

Table S2. Components of PGE currents remaining after elimination of the $\sigma_{zxx}^{\text{Re}}$ term (only the *z* components are modified in comparison to Table 1 in the main text).

| Ex. / Det. | $x$: S | $y$: P | $z$: P |
| --- | --- | --- | --- |
| S | 0 | $\text{0}$ | $0$ |
| P | 0 | $E_{0}^{2}\left\vert t_{p} \right\vert^{2}\sigma_{xxz}^{\mathrm{Re}}\sin2\theta$ | $E_{0}^{2}\left\vert t_{p} \right\vert^{2}\sigma_{zzz}^{\mathrm{Re}}\sin^{2} \theta$ |
| R | $E_{0}^{2}\left\vert t_{s}t_{p} \right\vert\left( \sigma_{xxz}^{\mathrm{Im}}\cos\delta-\sigma_{xxz}^{\mathrm{Re}}\sin\delta\right)\sin\theta$ | $\frac{1}{2}E_{0}^{2}\left\vert t_{p} \right\vert^{2}\sigma_{xxz}^{\mathrm{Re}}\sin2\theta$ | $\frac{1}{2}E_{0}^{2}\left\vert t_{p} \right\vert^{2}\sigma_{zzz}^{\mathrm{Re}}\sin^{2} \theta$ |
| L | $-E_{0}^{2}\left\vert t_{s}t_{p} \right\vert\left( \sigma_{xxz}^{\mathrm{Im}}\cos\delta-\sigma_{xxz}^{\mathrm{Re}}\sin\delta\right)\sin\theta$ | $\frac{1}{2}E_{0}^{2}\left\vert t_{p} \right\vert^{2}\sigma_{xxz}^{\mathrm{Re}}\sin2\theta$ | $\frac{1}{2}E_{0}^{2}\left\vert t_{p} \right\vert^{2}\sigma_{zzz}^{\mathrm{Re}}\sin^{2} \theta$ |
